# Supplementary material for: Multi-Stress Induction of the Mycobacterium tuberculosis MbcTA Bactericidal Toxin-Antitoxin System
Source: Toxins (Basel). 2020 May 16;12(5):329. doi: 10.3390/toxins12050329 (PMC7291234; doi:10.3390/toxins12050329)
Supplement: Supplementary file 1 [file toxins-12-00329-s001.pdf]

Supplementary Materials: Multi-stress induction of the *Mycobacterium tuberculosis* MbcTA bactericidal toxin-antitoxin system

Kanchiyaphat Ariyachaokun, Anna D. Grabowska, Claude Gutierrez and Olivier Neyrolles

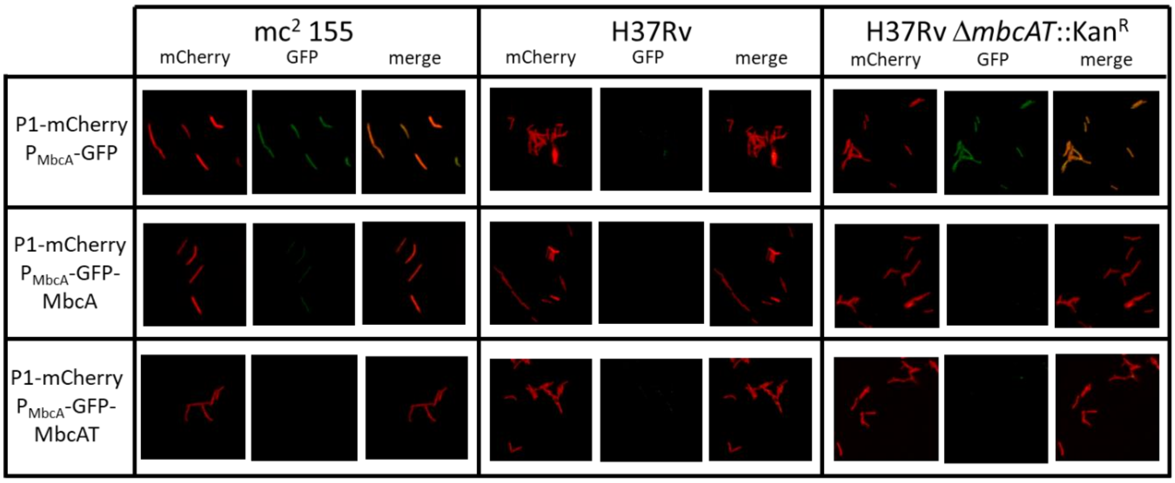

Figure S1. Auto-repression of the *mbcAT* operon expression in *M. smegmatis and M. tuberculosis*. Wild type *M. smegmatis* mc² 155 (left), wild type *M. tuberculosis* H37Rv (center) or mutant *M. tuberculosis* H37Rv Δ*mbcTA*::Kan^R (right) harboring plasmid pGMCS-P1-mCherry-P$_{mbcA}$GFP, pGMCS-P1-mCherry-P$_{mbcA}$GFP-*mbcA* or pGMCS-P1-mCherry-P$_{mbcA}$GFP-*mbcAT*, as indicated, were grown to exponential phase in complete 7H9 medium supplemented with streptomycin. Samples of the cells were fixed with 4% *p*-formaldehyde and observed in fluorescence microscopy using large field leica DMIRB, at magnification of 630×.

Table S1. Primers used for plasmid constructions.

Construction /DNA Fragment	Template	Primers	Sequence 5′-3′
**pEN41A-P1-GFP**			
P1	pEN12A-P1	Fw-GRFP-SD-P1	GCTCACCATGGTGAATCCTCCTCTCATTTCGGGCGGCGAATCTC
		Rev-B1-P1	GGGGACTGCTTTTTTGTACAAACTTGGGCGGCACGATCCGCGACGTG
GFP ORF	pLAM12:: GFP	Fw-B4-GFP	GGGGACAACTTTGTATAGAAAAGTTGTTACTTGTACAGCTCGTCCATG
		Rv-SD-GRFP	AGGAGGATTCACCATGGTGAGCAAGGGCGAGGAG
**pEN41A-P1-mCherry**			
P1	pEN12A-P1	Fw-GRFP-SD-P1	GCTCACCATGGTGAATCCTCCTCTCATTTCGGGCGGCGAATCTC
		Rv-B1-P1	GGGGACTGCTTTTTTGTACAAACTTGGGCGGCACGATCCGCGACGTG
mCherry ORF	pLAM12:: mCherry	Fw-B4-GRFP	GGGGACAACTTTGTATAGAAAAGTTGTTACTTGTACAGCTCGTCCATGCCG
		Rv-SD-GRFP	AGGAGGATTCACCATGGTGAGCAAGGGCGAGGAG
**pEN41A-P1-mTurquoise**			
P1	pEN12A-P1	Fw-mTurq-SD-P1	GCTGACCATGGTGAATCCTCCTCTCATTTCGGGCGGCGAATCTC
		Rv-B1-P1	GGGGACTGCTTTTTTGTACAAACTTGGGCGGCACGATCCGCGACGTG
mTurquoise ORF	pJYB 240-mTurquoise	Fw-B4-mTurq	GGGGACAACTTTGTATAGAAAAGTTGTTACTTGTAGAGTTCGTCCATGCCG
		Rv-SD-mTurq	AGGAGGATTCACCATGGTCAGCAAGGGTGAGGAACTC
**pEN41A-P1-mVenus**			
P1	pEN12A-P1	Fw-GRFP-SD-P1	GCTCACCATGGTGAATCCTCCTCTCATTTCGGGCGGCGAATCTC
		Rv-B1-P1	GGGGACTGCTTTTTTGTACAAACTTGGGCGGCACGATCCGCGACGTG
mVenus ORF	pJYB 234-mVenus	Fw-B4-GFP	GGGGACAACTTTGTATAGAAAAGTTGTTACTTGTACAGCTCGTCCATG
		Rv-SD-GRFP	AGGAGGATTCACCATGGTGAGCAAGGGCGAGGAG
**pEN12A-P*mbcA*-GFP**			
P*mbcA*(261)	H37Rv DNA	Fw-B1-PmbcA	GGGGACAAGTTTGTACAAAAAAGCAGGCTGCTGCTGGCTCCCAGTAGAC
		Rv-GRFP-PmbcA	GCTCACCATGGTGAATCCTCCTCCGGGCAGCCTGTCTTCCTTTTG
GFP ORF	pLAM12::GFP	Fw-PmbcA-GRFP	CAAAAGGAAGACAGGCTGCCCGGAGGAGGATTCACCATGGTGAGCAAGGGCGAGGAG
		Rv-B2-GRFP	GGGGACCACTTTGTACAAGAAAGCTGGGTTTACTTGTACAGCTCGTCCATG
**pEN12A-P*mbcA*-mCherry**			

$P_{mbcA(261)}$	H37Rv DNA	Fw-B1-P_{mbcA}	GGGGACAAGTTTGTACAAAAAAGCAG GCTGCTGCTGGCTCCCAGTAGAC
		Rv-GRFP-P_{mbcA}	GCTCACCATGGTGAATCCTCCTCCGGG CAGCCTGTCTTCCTTTTG
GFP ORF	pLAM12:: mCherry	Fw-P_{mbcA}-GRFP	CAAAAGGAAGACAGGCTGCCCGGAG GAGGATTCACCATGGTGAGCAAGGGC GAGGAG
		Rv-B2-GRFP	GGGGACCACTTTGTACAAGAAAGCTG GGTTTACTTGTACAGCTCGTCCATG
**pEN12A-P_{mbcA}-mTurquoise**			
P_{mbcA}(261)	H37Rv DNA	Fw-B1-P_{mbcA}	GGGGACAAGTTTGTACAAAAAAGCAG GCTGCTGCTGGCTCCCAGTAGAC
		Rv-mTurq-P_{mbcA}	GCTGACCATGGTGAATCCTCCTCCGGG CAGCCTGTCTTCCTTTTG
mVenus ORF	pJYB 234- mTurquoise	Fw-P_{mbcA}-mTurq	CAAAAGGAAGACAGGCTGCCCGGAG GAGGATTCACCATGGTCAGCAAGGGT GAGGAACTC
		Rv-B2-mTurq	GGGGACCACTTTGTACAAGAAAGCTG GGTTTACTTGTAGAGTTCGTCCATGCC G
**pEN12A-P_{mbcA}-mVenus**			
$P_{mbcA(261)}$	H37Rv DNA	Fw-B1-P_{mbcA}	GGGGACAAGTTTGTACAAAAAAGCAG GCTGCTGCTGGCTCCCAGTAGAC
		Rv-GRFP-P_{mbcA}	GCTCACCATGGTGAATCCTCCTCCGGG CAGCCTGTCTTCCTTTTG
mVenus ORF	pJYB 234- mVenus	Fw-P_{mbcA}-GRFP	CAAAAGGAAGACAGGCTGCCCGGAG GAGGATTCACCATGGTGAGCAAGGGC GAGGAG
		Rv-B2-GRFP	GGGGACCACTTTGTACAAGAAAGCTG GGTTTACTTGTACAGCTCGTCCATG
**pEN12A-$P_{mbcA(103)}$-GFP**			
$P_{mbcA(103)}$-GFP	pEN12A- $P_{mbcA(261)}$-GFP	Fw2-B1-P_{mbcA}	GGGGACAAGTTTGTACAAAAAAGCAG GCTCCGAAACTGAATGCCCATCG
		Rv-B2-GRFP	GGGGACCACTTTGTACAAGAAAGCTG GGTTTACTTGTACAGCTCGTCCATG
**pEN12A-$P_{mbcA(239)}$-GFP**			
$P_{mbcA(239)}$	pEN12A- $P_{mbcA(261)}$-GFP	Fw-B1-P_{mbcA}	GGGGACAAGTTTGTACAAAAAAGCAG GCTGCTGCTGGCTCCCAGTAGAC
		Rv-GRFP-P_{mbcA}	GCTCACCATGGTGAATCCTCCTCCCTA TTGATTTGTCATGTATTATGACACGAA CCGAGG
GFP ORF	pLAM12::GFP	Fw2-P_{mbcA}-GFP	ACATGACAAATCAATAGGGAGGAGGA TTCACCATGGTGAGCAAGGGCGAGGA G
		Rv-B2-GRFP	GGGGACCACTTTGTACAAGAAAGCTG GGTTTACTTGTACAGCTCGTCCATG
**pEN12A-$P_{mbcA(239\text{-}G)}$-GFP**			
$P_{mbcA(239\text{-}G)}$	pEN12A- $P_{mbcA(261)}$-GFP	Fw-B1-P_{mbcA}	GGGGACAAGTTTGTACAAAAAAGCAG GCTGCTGCTGGCTCCCAGTAGAC

		Rv3-GFP-P_{mbcA}	GCTCACCATGGTGAATCCTCCTCCCTA TTGATTTGTCATGTGTTATGACACGAA CCGAGG
GFP ORF	pLAM12:: GFP	Fw2-P_{mbcA}-GFP	ACATGACAAATCAATAGGGAGGAGGA TTCACCATGGTGAGCAAGGGCGAGGA G
		Rv-B2-GRFP	GGGGACCACTTTGTACAAGAAAGCTG GGTTTACTTGTACAGCTCGTCCATG
**pGMCS-P1-mCherry-$P_{mbcA(Mut1)}$-GFP-*mbcA***			
Mutated linear plasmid	pGMCS-P1-mCherry-$P_{mbcA(261)}$-*mbcA*	Inf-P*mbcA*-right	GGCTGCCCGGAGGAGGATTC
		Inf-P*mbcA*-Mut1-left	CTCCTCCGGGCAGCCTGTCTTCCTACG ATCTATTGATTTGTCATG
**pGMCS-P1-mCherry-$P_{mbcA(Mut2)}$-GFP-*mbcA***			
Mutated linear plasmid	pGMCS-P1-mCherry-$P_{mbcA(261)}$-*mbcA*	Inf-P*mbcA*-right	GGCTGCCCGGAGGAGGATTC
		Inf-P*mbcA* Mut2-left -	CTCCTCCGGGCAGCCCACCTTCCTTTT GTCTATTGATTTG
**pGMCS-P1-mCherry-$P_{mbcA(Mut1)}$-GFP**			
Mutated linear plasmid	pGMCS-P1-mCherry-$P_{mbcA(Mut1)}$-*mbcA*	Inf-del*mbcA*-right	GGGAGTGTTCGTGTGAGCTG
		Inf-del*mbcA*-left	CACACGAACACTCCCGAGGCGAGCAC ATTTACTCCC
**pGMCS-P1-mCherry-$P_{mbcA(Mut2)}$-GFP**			
Mutated linear plasmid	pGMCS-P1-mCherry-$P_{mbcA(Mut2)}$-*mbcA*	Inf-del*mbcA*-right	GGGAGTGTTCGTGTGAGCTG
		Inf-del*mbcA*-left	CACACGAACACTCCCGAGGCGAGCAC ATTTACTCCC
**pGMCS-P1-mCherry-$P_{mbcA(261)}$-*mbcA*(R33E)**			
Mutated linear plasmid	pGMCS-P1-mCherry-$P_{mbcA(261)}$-*mbcA*	Inf-*mbcA*-R33E-right	GTCGATGCCTCGCCG**GAG**TCCGTGGC GCGATGGACCGC
		Inf-*mbcA*-R33E-left	CGGCGAGGCATCGACGATG
**pGMCS-P1-mCherry-$P_{mbcA(261)}$-*mbcA*(R33E)-*mbcT***			
Mutated linear plasmid	pGMCS-P1-mCherry-$P_{mbcA(261)}$-*mbcAT*	Inf-*mbcA*-R33E-right	GTCGATGCCTCGCCG**GAG**TCCGTGGC GCGATGGACCGC
		Inf-*mbcA*-R33E-left	CGGCGAGGCATCGACGATG
**pGMCS-P1-mCherry-$P_{mbcA(261)}$-*mbcA*(R37E)**			
Mutated linear plasmid	pGMCS-P1-mCherry-$P_{mbcA(261)}$-*mbcA*	Inf-*mbcA*-R37E-right	CCGCGTTCCGTGGCG**GAG**TGGACCGC AGGTCAGGTGG
		Inf-*mbcA*-R37E-left	CGCCACGGAACGCGGCGAG

Table S2. Plasmids used in this work.

Name	Reference/Origin	Construction/Properties
pDE43-MCS	D. Schnappinger (Weill Cornell Medical College, New York, USA)	Destination vector for Gateway cloning. Strepto^R
pDO221A	D. Schnappinger	Destination vector to construct pEN12 entry plasmids for Gateway cloning. Amp^R
pDO23A	D. Schnappinger	Destination vector to construct pEN23 entry plasmids for Gateway cloning. Amp^R
pDO41A	D. Schnappinger	Destination vector to construct pEN41 entry plasmids for Gateway cloning. Amp^R
pEN12A-P1	D. Schnappinger	Middle entry clone for Gateway cloning. Template for amplification of P1 promoter. Amp^R
pEN12A-P606Pld	D. Schnappinger	Middle entry clone for Gateway cloning. Amp^R
pEN12A-P606	D. Schnappinger	Middle entry clone for Gateway cloning. Amp^R
pEN12A-P$_{mbcA}$-reporter	This study	Gateway cloning into pDO221A. Middle entry clone for Gateway cloning. Reporter can be GFP, mCherry, mTurquoise or mVenus. Amp^R
pEN23A-Mlu1	D. Schnappinger	Empty 5′ entry clone for Gateway cloning. Amp^R
pEN23A-*mbcT*	[1]	5′ entry clone for Gateway cloning. Amp^R
pEN23A-*mbcAT*	[1]	5′ entry clone for Gateway cloning. Amp^R
pEN41-TetR	D. Schnappinger	3′ entry clone for Gateway cloning. Amp^R
pEN41A-P1-reporter	This study	Gateway cloning into pDO41A. 3′ entry clone for Gateway cloning. Reporter can be GFP, mCherry, mTurquoise or mVenus. Amp^R
pGMCS-P1-mCherry-P$_{mbcA}$-GFP	This study	Gateway cloning. Reporter of P$_{mbcA}$ expression (261-bp Fw1/Rv1 DNA fragment). Integrates at the L5 mycobacteriophage integration site in mycobacteria. Strepto^R
pGMCS-P1-mCherry-P$_{mbcA}$-GFP-*mbcA*	This study	Gateway cloning. Reporter of P$_{mbcA}$ expression (261-bp Fw1/Rv1 DNA fragment). Integrates at the L5 mycobacteriophage integration site in mycobacteria. Strepto^R
pGMCS-P1-mCherry-P$_{mbcA}$-GFP-*mbcAT*	This study	Gateway cloning. Reporter of P$_{mbcA}$ expression (261-bp Fw1/Rv1 DNA fragment). Integrates at the L5 mycobacteriophage integration site in mycobacteria. Strepto^R
pGMCS-P1-mCherry-P$_{mbcA}$-GFP-*mbcA*(R33E)	This study	Site directed mutagenesis of pGMCS-P1-mCherry-P$_{mbcA}$-GFP-*mbcA*. Strepto^R
pGMCS-P1-mCherry-P$_{mbcA}$-GFP-*mbcA*(R37E)	This study	Site directed mutagenesis of pGMCS-P1-mCherry-P$_{mbcA}$-GFP-*mbcA*. Strepto^R
pGMCS-P1-mCherry-P$_{mbcA}$-GFP-*mbcA*(R33E)-*mbcT*	This study	Site directed mutagenesis of pGMCS-P1-mCherry-P$_{mbcA}$-GFP-*mbcAT*. Strepto^R

pGMCS-P1-mCherry-$P_{mbcA(103)}$-GFP	This study	Gateway cloning. Reporter of P_{mbcA} expression (103-bp Fw2/Rv1 DNA fragment). Integrates at the L5 mycobacteriophage integration site in mycobacteria. $Strepto^R$
pGMCS-P1-mCherry-$P_{mbcA(103)}$-GFP-*mbcA*	This study	Gateway cloning. Reporter of P_{mbcA} expression (103-bp Fw2/Rv1 DNA fragment). Integrates at the L5 mycobacteriophage integration site in mycobacteria. $Strepto^R$
pGMCS-P1-mCherry-$P_{mbcA(239)}$-GFP	This study	Gateway cloning. Reporter of P_{mbcA} expression (239-bp Fw1/Rv2 DNA fragment). Integrates at the L5 mycobacteriophage integration site in mycobacteria. $Strepto^R$
pGMCS-P1-mCherry-$P_{mbcA(239)}$-GFP-*mbcA*	This study	Gateway cloning. Reporter of P_{mbcA} expression (239-bp Fw1/Rv2 DNA fragment). Integrates at the L5 mycobacteriophage integration site in mycobacteria. $Strepto^R$
pGMCS-P1-mCherry-$P_{mbcA(239\text{-}G)}$-GFP	This study	Gateway cloning. Reporter of P_{mbcA} expression (239-bp Fw1/Rv3 DNA fragment). Integrates at the L5 mycobacteriophage integration site in mycobacteria. $Strepto^R$
pGMCS-P1-mCherry-$P_{mbcA(Mut1)}$-GFP-*mbcA*	This study	Site-directed mutagenesis of pGMCS-P1-mCherry-P_{mbcA}-GFP-*mbcA*. $Strepto^R$
pGMCS-P1-mCherry-$P_{mbcA(Mut1)}$-GFP	This study	Site-directed mutagenesis of pGMCS-P1-mCherry-$P_{mbcA(Mut1)}$-GFP-*mbcA*. $Strepto^R$
pGMCS-P1-mCherry-$P_{mbcA(Mut2)}$-GFP-*mbcA*	This study	Site-directed mutagenesis of pGMCS-P1-mCherry-P_{mbcA}-GFP-*mbcA*. $Strepto^R$
pGMCS-P1-mCherry-$P_{mbcA(Mut2)}$-GFP	This study	Site-directed mutagenesis of pGMCS-P1-mCherry-$P_{mbcA(Mut2)}$-GFP-*mbcA*. $Strepto^R$
pGMCS-P1-mTurquoise-P_{mbcA}-mVenus	This study	Gateway cloning. Reporter of P_{mbcA} expression. Integrates at the L5 mycobacteriophage integration site in mycobacteria. $Strepto^R$
pGMCS-TetR-P1-*mbcT*	[1]	Allow tetracycline-inducible expression of *mbcT*. Integrates at the L5 mycobacteriophage integration site in mycobacteria. $Strepto^R$
pGMCS-TetR-P606Pld-*mbcT*	This study	Allow tetracycline-inducible expression of *mbcT*. Integrates at the L5 mycobacteriophage integration site in mycobacteria. $Strepto^R$
pGMCS-TetR-P606-*mbcT*	This study	Allow tetracycline-inducible expression of *mbcT*. Integrates at the L5 mycobacteriophage integration site in mycobacteria. $Strepto^R$
pJYB 240-mTurq	J.Y. Bouet & J. Rech (LMGM, Toulouse, France)	Template for amplification of *mTurquoise* by PCR
pJYB 234-mVenus	J.Y. Bouet & J. Rech	Template for amplification of *mVenus* by PCR
pLAM12::GFP	[39]	Template for amplification of *egfp* by PCR.
pLAM12::mCherry	[39]	Template for amplification of *mCherry* by PCR
